# Genome-Wide Identification of the *TaBON* Gene Family and Its Role in Wheat Pathogen Response

**DOI:** 10.3390/biology15090704

**Published:** 2026-04-30

**Authors:** Yanzhen Wang, Yongtao Zhao, Jialu Li, Xia Liu, Menglin Lei

**Affiliations:** 1 Center for Agricultural Genetic Resources Research, Shanxi Agricultural University, Taiyuan 030031, China; 2 Luohe Academy of Agricultural Sciences, Luohe 462300, China; 3 College of Agriculture, Shanxi Agricultural University (Institute of Crop Sciences), Taigu 030801, China

**Keywords:** wheat, *TaBON* gene family, expression analysis, pathogen response

## Abstract

The BONZAI/BON (copine) gene family encodes evolutionarily conserved proteins that modulate the balance between plant defense responses and growth. However, comprehensive identification and functional exploration of *BON* members have remained largely lacking in wheat. In this study, we identified 10 *Triticum aestivum BON* (*TaBON*) members, which are unevenly distributed across seven wheat chromosomes. Phylogenetic analysis clustered these TaBON proteins into two distinct groups. Gene structure and conserved motif analyses revealed high evolutionary conservation within the *TaBON* family. *Cis*-acting element analysis revealed that the promoter regions of *TaBON* genes are enriched with elements responsive to hormones, abiotic stress, and biotic defense signals. Expression profiling further demonstrated distinct transcriptional patterns of *TaBON* genes in response to infections by *Puccinia striiformis* f. sp. *tritici* (stripe rust), *Blumeria graminis* f. sp. *tritici* (powdery mildew), *Fusarium graminearum*, and *Zymoseptoria tritici*. Overall, this study presents a comprehensive analysis of the *TaBON* members and provides valuable molecular information for understanding its role in disease resistance.

## Simple Summary

This study identified 10 *TaBON* genes in wheat and systematically analyzed their evolutionary, physicochemical properties, as well as gene and protein structures, and regulatory elements. Furthermore, expression pattern analysis showed that various *TaBON* genes display distinct transcriptional profiles when wheat plants are infected by four major pathogens (stripe rust, powdery mildew, *Fusarium graminearum*, and *Zymoseptoria tritici*). Together, these findings lay a foundation for further exploration of the functional roles of *TaBON* genes and their potential implications in processes related to wheat disease resistance.

## 1. Introduction

The copine protein family, designated as BONZAI (*BON*) in plant systems, represents a phylogenetically conserved group of calcium-regulated phospholipid-binding proteins that are widely distributed across eukaryotic lineages [1]. Structurally, these proteins are characterized by a tandem pair of C_2_ domains at the N-terminus, which endow them with calcium-dependent membrane-binding capabilities, and a C-terminal von Willebrand factor A (vWFA) domain that mediates essential protein–protein interactions for integrating into cellular signaling networks [2]. This unique modular architecture enables copines to act as versatile regulators in core cellular processes, including vesicular trafficking, signal transduction, and the modulation of cell proliferation, differentiation, and apoptosis [3,4].

To date, genome-wide identification and characterization of the *BON* gene family has been conducted in several plant species, including *Arabidopsis thaliana* [5], rice (*Oryza sativa*) [6], and maize (*Zea mays*) [7], which have revealed the diversity, structural features, and functional roles of these genes. In *Arabidopsis thaliana*, copine genes were named BONZAI (*BON*) because mutants of these genes exhibit a dwarf phenotype. BON proteins regulate plant signaling pathways through multiple molecular mechanisms. Firstly, BON proteins directly interact with SERK kinases in the brassinosteroid (BR) signaling pathway, thereby modulating BR signal transduction. Studies in *Arabidopsis* and maize have shown that loss of BON function impairs BR signaling, leading to phenotypes such as dwarfism and reduced BR sensitivity [7]. Secondly, BON1 dynamically regulates plant immunity and development via protein–protein interactions and post-translational modifications. It synergistically interacts with PUB13 to coordinate flowering and defense responses, while reversible S-acylation mediated by PAT14 and ABAPT11 fine-tunes immune receptor internalization through the clathrin adaptor CLC3 [8,9]. Additionally, BON proteins are involved in biotic stress responses and function as negative regulators of NLR-mediated immune signaling [10]. In rice, *OsBON1* and *OsBON3* function as negative regulators of broad-spectrum disease resistance [6].

As a cornerstone of global food security, wheat is constantly threatened by fungal diseases such as stripe rust [11], powdery mildew [12], and *Fusarium graminearum* [13], which cause substantial annual yield losses worldwide [14]. Although conventional breeding has deployed major resistance genes, the rapid evolution of pathogen virulence frequently undermines the durability of resistant varieties. This necessitates the dissection of core immune regulatory networks to support sustainable crop improvement [15]. Previous studies have revealed that wheat copine genes *TaBON1* and *TaBON3*, which negatively regulate powdery mildew resistance, influence immune responses by suppressing hydrogen peroxide accumulation and cell death pathways [16]. However, research on the wheat *BON* gene family remains in its infancy. Critical knowledge gaps remain, including an incomplete genome-wide identification of *TaBON* members, unclear chromosomal distribution and evolutionary relationships, and a lack of systematic analysis of their expression dynamics and functional diversification under diverse biotic stresses. Moreover, whether TaBON proteins employ post-translational modifications or epigenetic mechanisms to modulate wheat immune signaling awaits experimental validation. Therefore, comprehensive characterization of the *TaBON* gene family is not only essential to elucidate its role in wheat–pathogen interactions but also holds practical potential for developing broad-spectrum disease-resistant varieties [17].

In this research, we conducted a comprehensive genome-wide identification and functional analysis of the wheat *BON* gene family. Through integrated bioinformatic analyses, we systematically identified *TaBON* genes and elucidated their structural features, conserved motifs, phylogenetic evolution, chromosomal distribution, and collinearity relationships. Furthermore, by integrating transcriptomic data and RT-qPCR experiments, we examined the expression patterns of *TaBON* genes under various fungal stresses, including stripe rust, powdery mildew, *Fusarium graminearum*, and *Zymoseptoria tritici*. The results revealed significant functional differentiation among *TaBON* gene family members in response to different pathogen infections, providing new insights into the molecular mechanisms underlying wheat–pathogen interactions and offering potential target genes for wheat disease resistance breeding.

## 2. Materials and Methods

### 2.1. Experimental Materials and Treatment

The experimental materials used in this study was the wheat–*Thinopyrum ponticum* disomic alien substitution line CH10A5 [18]. Seeds were surface-sterilized with 1% (*v*/*v*) sodium hypochlorite for 10 min, rinsed thoroughly with sterile distilled water to remove residual disinfectant, and germinated in darkness at 25 °C for 24 h. Germinated seedlings were subsequently grown in a growth chamber under a 16 h light/8 h dark photoperiod (light intensity: 300 μmol·m^−2^·s^−1^) at 25 ± 1 °C. At the two-leaf stage, seedlings were inoculated with the stripe rust pathogen (*Puccinia striiformis* f. sp. *tritici*) race CYR34 using the spray inoculation method, and samples were collected at 0, 24, and 48 h post-inoculation (hpi), respectively. In a separate batch, seedlings at the same growth stage were inoculated with the powdery mildew strain E09 (*Blumeria graminis* f. sp. *tritici*) via the dusting method. Samples were collected at 0, 12, 24, and 48 hpi, respectively. All leaf tissues were immediately flash-frozen in liquid nitrogen and then stored at −80 °C for subsequent RNA extraction. All treatments were performed with three biological replicates to ensure reproducibility.

### 2.2. Identification and Physicochemical Property Analysis of the TaBON Members

Genomic data of common wheat (*Triticum aestivum* cv. Chinese Spring RefSeq v1.1), *Arabidopsis thaliana* (TAIR 10), rice (*Oryza sativa* Japonica, MSU 7.0), maize (*Zea mays*), and foxtail millet (*Setaria italica* v2.0), encompassing reference genome sequences, protein sequences, and GFF3 annotation files, were sourced from the Ensembl Plants database (https://plants.ensembl.org/index.html, accessed on 5 June 2025) [19]. Protein sequences of known BON members in *Arabidopsis thaliana* were retrieved from TAIR (https://www.arabidopsis.org/, accessed on 7 June 2025) and used as query templates for BLASTP 2.12.0+ (E-value < 1 × 10^−5^) searches against the protein databases of the aforementioned species using TBtools II (version 2.390) software [20]. Additionally, Hidden Markov Models (HMMs) corresponding to the conserved domain of the BON protein (PF07002) were downloaded from Pfam (http://pfam.xfam.org/, accessed on 10 June 2025), and the HMMER 3.3 tool was then employed to search the whole-genome protein sequences of all selected species against these HMM profiles with an E-value cutoff of <1 × 10^−10^. The candidate *BON* members were further validated via the NCBI Conserved Domain Database (NCBI-CDD) tool (https://www.ncbi.nlm.nih.gov/Structure/bwrpsb/bwrpsb.cgi, accessed on 10 June 2025) to confirm the presence of characteristic BON domains. *TaBON* family members were definitively identified by integrating results from BLASTP, HMMER, and NCBI-CDD analyses. Functional annotations of *TaBON* genes were verified using the Triticeae-GeneTribe database (http://wheat.cau.edu.cn/TGT/, accessed on 19 June 2025) [21]. *TaBON* members were renamed according to their chromosomal locations.

The protein sequences of the identified TaBON members were submitted to the online website ExPASy ProtParam (https://web.expasy.org/protparam/, accessed on 2 August 2025) to predict key physicochemical properties, including molecular weight, theoretical isoelectric point (pI), amino acid composition, instability index, and grand average of hydropathicity (GRAVY). Subcellular localization of TaBON proteins was predicted using online tools DeepLoc 2.1 (https://services.healthtech.dtu.dk/services/DeepLoc-2.1/, accessed on 5 August 2025) and CELLO (http://cello.life.nctu.edu.tw/, accessed on 5 August 2025).

### 2.3. Phylogenetic and Chromosomal Localization Analysis of TaBON Members

To clarify the evolutionary relationships of *BON* family members, full-length protein sequences of BON proteins from *Arabidopsis thaliana*, rice, maize, foxtail millet, and wheat were aligned using Clustal W in MEGA software (version 11.0.13). The alignment parameters were set as follows: Gap Opening Penalty = 15, Gap Extension = 6.66, DNA Weight Matrix = IUB) [22]. A phylogenetic tree was constructed using the Maximum Likelihood (ML) method with 1000 bootstrap replicates. The JTT substitution model was selected based on the characteristics of the protein sequences [23]. The phylogenetic tree was visualized and refined using the online Interactive Tree Of Life (iTOL) v7 tool (http://itol.embl.de/, accessed on 15 August 2025) [24]. Chromosomal locations of *TaBON* members were mapped using TBtools based on positional information extracted from the GFF3 annotation files.

### 2.4. Analysis of Conserved Motifs, Domains, and Gene Structures

The conserved motifs of the *TaBON* members were identified using the MEME Suite v5.0.5 (https://meme-suite.org/meme/tools/meme, accessed on 20 August 2025), with the maximum number of motifs set to 15 and other parameters kept at default settings [25]. The conserved domains were predicted using the Batch CD-Search tool on the NCBI platform (https://www.ncbi.nlm.nih.gov/Structure/bwrpsb/bwrpsb.cgi, accessed on 20 August 2025). The gene structures (exons, introns, UTRs) of *TaBON* members were determined based on the GFF3 annotation files using the “Gene Structure View (Advanced)” module in TBtools II v2.390. The visual integration of the phylogenetic tree, conserved motifs, domains, and gene structures was generated using TBtools.

### 2.5. Cis-Acting Elements Prediction Analysis of TaBONs

The promoter regions, defined as 2000 bp upstream of the translation start codon (ATG) of each *TaBON* gene, were extracted from WheatOmics 1.0 (http://wheatomics.sdau.edu.cn/, accessed on 25 August 2025) [26]. *Cis*-acting elements within these promoter sequences were predicted using the PlantCARE database (https://bioinformatics.psb.ugent.be/webtools/plantcare/html/, accessed on 6 September 2025) [27]. Elements related to plant growth and development, phytohormone response, and stress responses were screened and classified. The distribution of these *cis*-acting elements was visualized using TBtools v2.390.

### 2.6. Gene Duplication and Synteny Analysis of TaBONs

Gene duplication events (including tandem and segmental duplications) within the *TaBON* gene family were identified via One Step MCScanX tools in TBtools II v2.390 (E-value < 1 × 10^−10^) using wheat RefSeq v1.1 synteny data. The synteny analysis results were visualized using the built-in module of MCScanX [28].

### 2.7. Transcriptome-Based Analysis of TaBON Expression in Leaves in Response to Pathogen Infection

The RNA-seq expression data (Transcripts Per Million, TPM values) of *TaBON* members under three distinct biotic stresses were obtained from public wheat transcriptome databases WheatExp (https://www.wheat-expression.com/, accessed on 5 October 2025) [29,30]. Specifically, expression profiles in response to *Fusarium graminearum* infection were accessed from the ERP013829 dataset, while transcriptomic data related to *Zymoseptoria tritici* were retrieved from the study ERP009837 repository. Expression patterns were analyzed, and heatmaps were generated using TBtools II software (version 2.390) to visualize the transcriptional dynamics of *TaBON* genes, with colors in heatmaps indicating expression levels (red: high expression; blue: low expression).

### 2.8. RT-qPCR Analysis of TaBON Gene Expression Under Stripe Rust Infection

To investigate the expression dynamics of *TaBON* genes in response to powdery mildew and stripe rust infection, all ten identified *TaBON* genes were selected for reverse transcription quantitative PCR (RT-qPCR) analysis. Total RNA was extracted from wheat leaves collected post-inoculation with *Blumeria graminis* f. sp. *tritici* and *Puccinia striiformis* f. sp. *tritici*. In both experiments, the 0 hpi time point was used as the experimental control. Each time point included three independent biological replicates. The cDNA was synthesized and amplified on a QuantStudio™ 6 Flex Real-Time PCR System using TBGreen^®^ Premix Ex Taq™ II (Tli RNaseH Plus; Code No. RR820A, TaKaRa, Kusatsu, Japan). The housekeeping gene *TaActin* was used as an internal control for normalization. Relative expression levels were calculated using the 2^−ΔΔCT^ method. Statistical significance of differences among treatments was determined by one-way ANOVA followed by Duncan’s multiple range test (*p* < 0.05) in GraphPad Prism version 10.1.0. The expression analysis of each gene was based on qRT-PCR data obtained from three biological replicates and three technical replicates. All primers were synthesized by Shanghai Sangon Biotech (Shanghai, China), and their sequences are provided in Appendix A Table A1.

### 2.9. Subcellular Localization Analysis

Initial bioinformatics analysis predicted two potential subcellular localizations for TaBON6: the cytoplasm and the plasma membrane. To determine the subcellular localization of TaBON6, the full-length coding sequence (CDS) of *TaBON6* (excluding the stop codon) was amplified and fused in-frame to the pCAMBIA1302-EGFP vector, generating the TaBON-EGFP recombinant plasmid. This recombinant plasmid was transferred into *Agrobacterium strain GV3101*, and then co-transformed with a plasma membrane (PM)-localized mCherry marker (PM-mCherry) into *Nicotiana benthamiana* leaves. The plants were incubated in the dark for 48 h to allow protein expression. GFP fluorescence was observed using a confocal laser scanning microscopy (CLSM). Subcellular localization of the TaBON6 protein was determined by colocalization analysis of the GFP signal with the mCherry signal from the PM marker. The experiment was repeated three times independently to ensure reliability.

## 3. Results

### 3.1. Identification and Basic Physicochemical Properties of TaBON Members

Through the integrated analysis of BLASTP, HMMER, NCBI-CDD validation, and Triticeae-GeneTribe functional annotation, a total of 10 *TaBON* family members were identified, and then renamed according to their chromosomal locations (Supplementary Table S1). Bioinformatic analysis of the ten wheat *BON* family members revealed conserved physicochemical properties with notable inter-member variations (Table 1). The amino acid lengths of TaBON proteins ranged from 574 (TaBON5 and TaBON9) to 633 (TaBON4), with corresponding molecular weights (MW) of 62.96–69.25 kDa. Theoretical isoelectric points (pI) were predominantly acidic, with nine members clustering between 5.18 and 5.78, while TaBON10 exhibited a distinct basic pI of 8.63. All ten proteins displayed instability indices below 40, indicating stable protein configurations, with TaBON10 being the most stable (29.78) and TaBON4 the least (40.44). Aliphatic indices ranged from 83.63 to 93.2, suggesting high thermostability among all TaBON proteins. Grand average of hydropathicity (GRAVY) values were consistently negative (−0.211 to −0.122), demonstrating that all TaBON proteins are hydrophilic. Subcellular localization predictions indicated that all TaBON proteins localize to the membrane, establishing a consistent membrane-associated characteristic across the entire family. These analyses reveal the diversity in physicochemical properties among *TaBON* family members and provide a foundation for further functional characterization.

### 3.2. Phylogenetic Analysis and Chromosomal Localization of the BON Gene Family in Wheat

**Figure 1:**
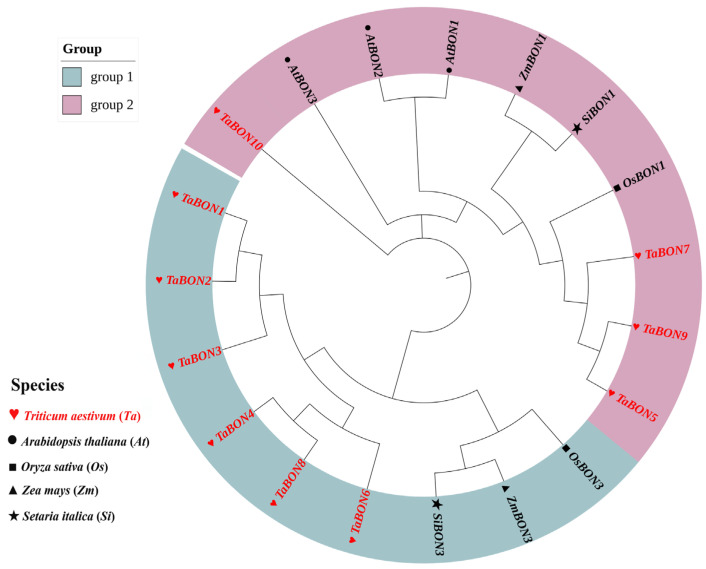
Phylogenetic analysis of BON proteins were from *Arabidopsis thaliana* (At), *Oryza sativa* (Os), *Zea mays* (Zm), and *Setaria italica* (Si) and *Triticum aestivum* (Ta). The phylogenetic tree was inferred using the Maximum Likelihood (ML) method with MEGA 11 software (version 11.0.13).

Phylogenetic analysis of the *BON* gene family across five plant species (*Triticum aestivum*, *Arabidopsis thaliana*, *Oryza sativa*, *Zea mays*, and *Setaria italica*) revealed distinct evolutionary relationships (Supplementary Table S2). The maximum-likelihood tree clearly divided the *BON* genes into two major clades (Group 1 and Group 2), indicating an early functional divergence within this gene family prior to the speciation of monocots and dicots (Figure 1). *TaBONs* were distributed across both groups, with closer phylogenetic relationships to their orthologs from monocot species (*O. sativa*, *Z. mays*, and *S. italica*) than to the dicot *A. thaliana.* Within the monocot lineage, *TaBON* genes frequently clustered with their putative orthologs from rice and maize, reflecting shared ancestry and potential functional conservation among grasses. This phylogenetic pattern suggests that the core functions mediated by the *BON* family were established early in plant evolution and have been largely retained in wheat.

**Figure 2:**
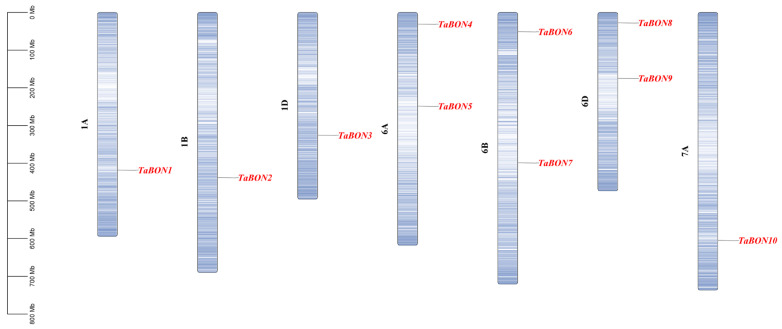
Chromosome distribution of *TaBON* members in wheat genome.

Chromosomal localization analysis of the ten *TaBON* genes revealed that they are distributed on chromosomes belonging to homologous groups 1, 6, and 7 of the wheat genome, specifically on chromosomes 1A, 1B, 1D, 6A, 6B, 6D and 7A (Figure 2). This distribution pattern highlights several important features. First, the *TaBON* genes are located on the A, B, and D subgenomes of wheat, reflecting the hexaploid nature of its genome. For instance, homologous genes are present on chromosomes 1A, 1B, 1D and 6A, 6B, 6D, consistent with the origin of hexaploid wheat from the hybridization of three diploid ancestors. However, only *TaBON10* is located on chromosome 7A, with no homologous genes detected on 7B or 7D, suggesting potential gene loss events on chromosomes 7B and 7D following polyploidization. Physical mapping also showed that some chromosomes (e.g., 1D) carry multiple *TaBON* genes, indicating that, in addition to whole-genome duplication associated with polyploidization, both tandem duplication (closely located on the same chromosome) and dispersed duplication (distributed across different chromosomes) events have collectively contributed to the expansion of the *BON* gene family in wheat.

### 3.3. Comparative Analysis of Gene Structure and Protein Features of TaBON Family Members

**Figure 3:**
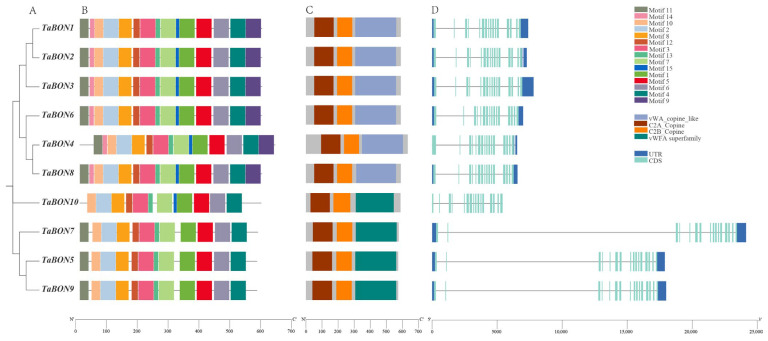
Comprehensive analysis of *TaBON* gene family members. (**A**) Phylogenetic tree showing evolutionary relationships. (**B**) MEME-based visualization of conserved motifs, with colored boxes representing specific conserved amino acid sequences. (**C**) Conserved domain architecture inferred from NCBI-CDD analysis, with color-coded boxes indicating phylogenetically conserved functional modules. (**D**) Gene structure analysis depicting exon–intron organization of *TaBON* members.

A phylogenetic tree constructed from the protein sequences shows the evolutionary relationships among the ten TaBON proteins (Figure 3A). The tree reveals distinct clustering, with *TaBON1*, *TaBON2,* and *TaBON3* forming one closely related subgroup, and *TaBON6*, *TaBON4*, and *TaBON8* grouping together. *TaBON10*, *TaBON7*, *TaBON5*, and *TaBON9* appear on separate branches, indicating greater divergence. This phylogenetic grouping suggests potential functional differentiation within the gene family.

To investigate the structural characteristics of the wheat *BON* gene family, a conserved motif analysis was conducted based on their phylogenetic relationships. Using the MEME suite, 15 conserved motifs were identified across the *TaBON* family (Figure 3B). Among these, 11 motifs were universally present in all members, suggesting they may encode core functional domains essential to the protein family. Phylogenetic grouping revealed distinct clade-specific motif patterns: Motif 9 and Motif 14 were exclusively found in Group I, while Motif 15 was largely absent in Group II, with the exception of *TaBON10*. These differences in motif composition indicate potential functional divergence among evolutionary clades, providing structural insights into the biological roles of the *TaBON* gene family in wheat.

The schematic representation of protein domains, predicted using Pfam, shows that all ten TaBON proteins contain the characteristic BON-associated domain (Figure 3C). All TaBON proteins contain the C2A and C2B domains at the N-terminus. The length and position of this core domain are largely conserved, confirming that all identified members belong to the *BON* family. Minimal variations in domain length are observed at the N- or C-termini of some proteins, which might influence subcellular localization or protein–protein interactions.

The illustrated exon–intron structure of the *TaBON* genes indicates a general conservation of gene organization within phylogenetic groups (Figure 3D). This conservation is evident within group 1 (e.g., *TaBON1/2/3*), where members share a similar number of exons and introns and have exons of comparable length. Conversely, structural organization varies more significantly between group 2. Further diversity was observed in the lengths of upstream/downstream untranslated regions (UTRs) and introns; this variation in non-coding regions may underlie differences in gene regulation and expression patterns among gene family members.

### 3.4. TaBON Gene Duplication and Collinearity Analysis

**Figure 4:**
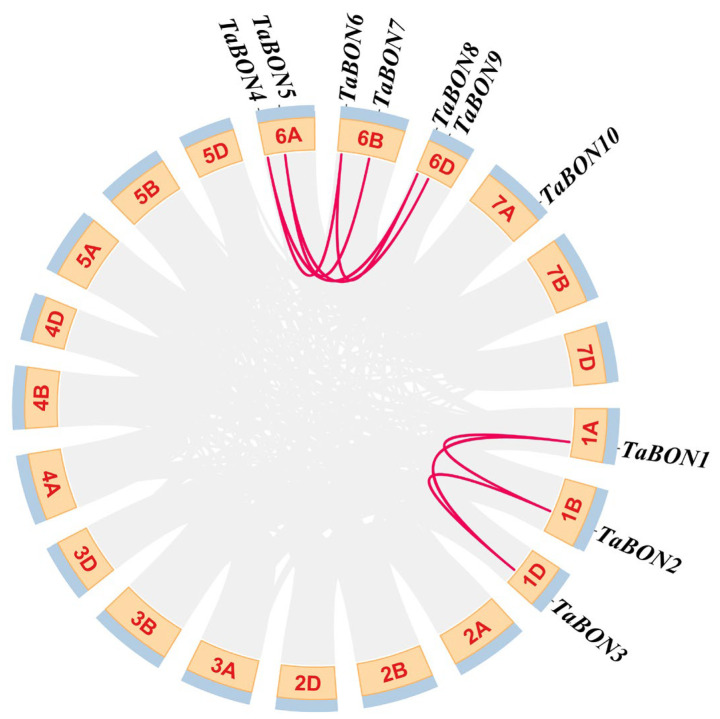
Genomic collinearity analysis of *TaBON* gene family members. Red lines indicate gene pairs with collinearity relationships, numbers within the boxes represent chromosomes, and light blue boxes denote gene density.

To investigate the expansion mechanisms of the *TaBON* gene family, we systematically analyzed duplication events across the wheat subgenomes. The circular map visualization revealed two distinct duplication clusters (Figure 4). The first cluster was localized on homoeologous chromosomes 6A, 6B, and 6D, suggesting a recent duplication event likely associated with post-polyploidization divergence. In contrast, the second cluster containing *TaBON1*, *TaBON2*, and *TaBON3* on chromosomes 1A, 1B, and 1D displayed syntenic conservation, indicating an ancient duplication predating wheat subgenome divergence. Notably, *TaBON10* on chromosome 7B remained as a singleton without proximal paralogs, potentially representing a functionally specialized member retained for unique adaptive roles. Overall, this analysis demonstrates that both ancient and recent duplication events have substantially shaped the *TaBON* family architecture, thereby generating functional redundancy while simultaneously providing the genetic substrate for neofunctionalization and enhanced stress resilience in wheat.

To further elucidate the evolutionary dynamics and selective pressures affecting the duplicated *TaBON* genes, we calculated the ratios of *Ka* and *Ks* substitutions for the 8 duplicated *TaBON* gene pairs (Supplementary Table S3). The results indicated that Ka/Ks values for the 8 gene pairs ranged from 0.0476 to 0.3441. Since these Ka/Ks values were all less than 1, this suggests that the *TaBON* gene family has experienced strong purifying selection pressure during evolution.

**Figure 5:**
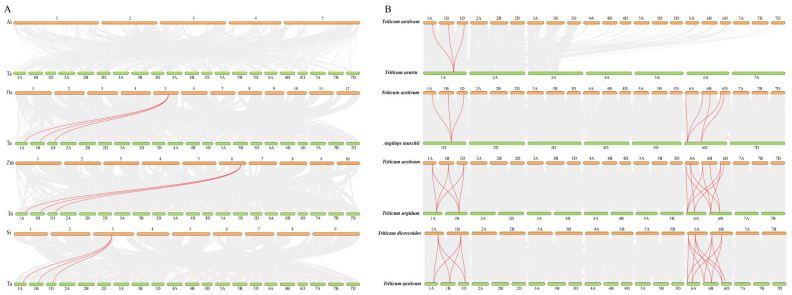
Interspecific collinearity analysis of *BON* gene family members. (**A**) Collinearity analysis between wheat and *Arabidopsis thaliana*, rice, maize, and foxtail millet. Species abbreviations: Ta, *Triticum aestivum*; At, *Arabidopsis thaliana*; Os, *Oryza sativa*; Zm, *Zea mays*; Si, *Setaria italica*. (**B**) Genomic collinearity analysis among hexaploid wheat and its diploid and tetraploid progenitors. Red lines indicate collinear gene pairs between wheat (Ta) and other plant species.

To elucidate the evolutionary relationships of *TaBON* genes, we conducted collinearity analysis between wheat and *Arabidopsis thaliana*, rice, maize, and foxtail millet (Figure 5A). No collinear gene pairs were detected between *Arabidopsis thaliana* and wheat, whereas *TaBON1*, *TaBON2*, and *TaBON3* exhibited conserved synteny with orthologs in rice, maize, and foxtail millet, suggesting functional conservation following the monocot/dicot divergence. In addition, we performed collinearity analysis between hexaploid wheat and its diploid progenitors (*Triticum urartu* and *Aegilops tauschii*) and tetraploid ancestor (*T. turgidum* and *T. dicoccoides*) (Figure 5B). The syntenic map revealed that most *TaBON* genes maintained robust collinear relationships with their ancestral orthologs, indicating that the BON family was largely preserved through two major polyploidization events of wheat. These findings collectively demonstrate that while certain *TaBON* genes have remained functionally conserved across monocots, the entire family has been under strong selective pressure to maintain gene dosage and function during wheat polyploid evolution.

### 3.5. Analysis of Cis-Acting Elements in TaBON Gene Promoters

**Figure 6:**
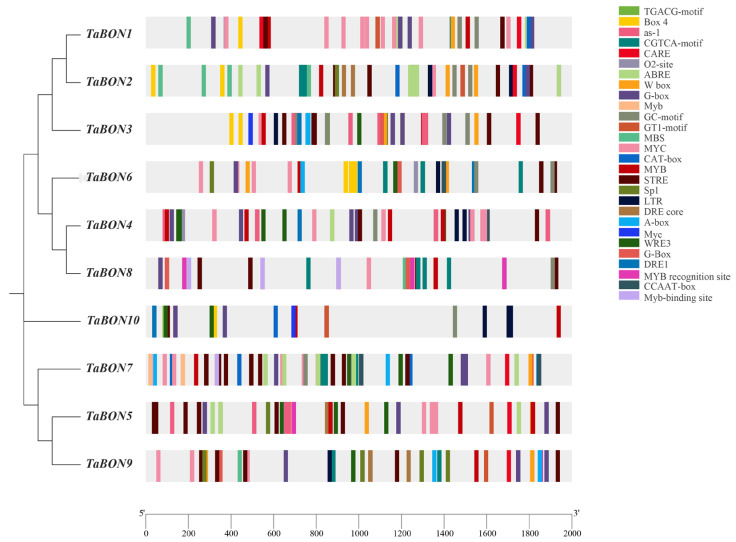
Analysis of *cis*-acting elements in *TaBON* members. Distinct *cis*-acting elements are represented by boxes in different colors, as defined in the legend on the right.

To elucidate the molecular basis of *TaBON* gene transcriptional regulation, this study systematically analyzed the 2000 bp promoter regions upstream of the 10 *TaBON* genes, identifying a specific distribution pattern of *cis*-acting elements and their systematic clustering characteristics (Figure 6). A total of 451 *cis*-acting elements were identified, encompassing various types including light-responsive elements, hormone-responsive elements, abiotic stress-responsive elements, and transcription factor binding sites. Among all the *cis*-acting elements identified, the G-box element is the most abundant, with a total of 45 occurrences, followed by ABRE (40) and STRE (40). Other key elements included methyl jasmonate and auxin motifs (33 each), MYB/MYC binding sites (38/37), stress-responsive LTR (10), and defense-related W box (12) and WRE3 (16). This composition implies that *TaBON* genes might contribute to adaptive responses influencing wheat disease resistance.

Importantly, element composition varied substantially among individual *TaBON* genes (Supplementary Table S4). *TaBON4* and *TaBON7* were enriched in MYB, MYC, and STRE binding sites, whereas *TaBON2*, *TaBON3* and *TaBON9* exhibited higher densities of light-responsive elements. These differences reflect potential divergence in regulatory mechanisms and biological functions among the *TaBON* genes. The abundance of stress and hormone-responsive elements in their promoters indicates that this gene family may be crucial for integrating various environmental signals to regulate stress adaptation and growth processes in wheat.

### 3.6. Expression Profiling of the TaBON Gene Family in Response to Four Biotic Stresses

**Figure 7:**
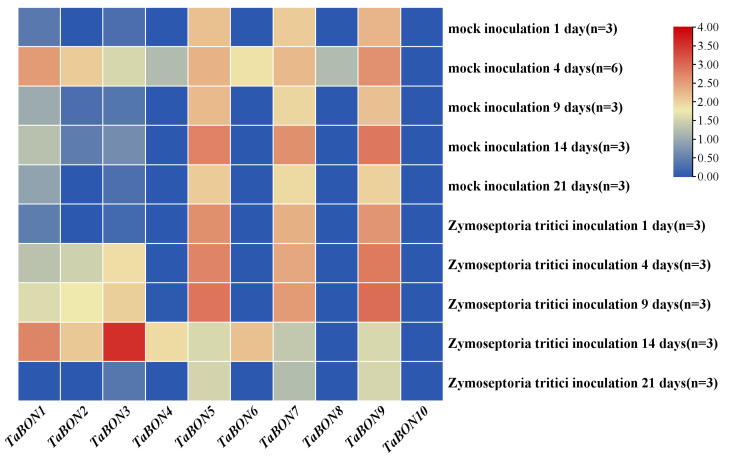
Distinct Expression Pattern of *TaBON* genes during *Zymoseptoria tritici* Infection.

The *TaBON* gene family in wheat displayed significant expression differentiation and temporal dynamics in response to *Zymoseptoria tritici* (Zt) infection (Figure 7). Under mock inoculation conditions, *TaBON5*, *TaBON7*, and *TaBON9* exhibited sustained high expression, while the remaining *TaBON* genes showed significant upregulation specifically at 4 days post-inoculation (dpi) and subsequently maintained relatively low basal expression levels during later stages, except for *TaBON10*. Following Zt infection, distinct induction patterns emerged among the family members. The heatmap visually revealed that *TaBON2* and *TaBON3* were strongly and specifically upregulated during early infection (1 and 4 dpi), whereas *TaBON4* and *TaBON6* displayed marked upregulation at the later infection stage (14 dpi). Overall, the *TaBON* gene family exhibited heterogeneity in both the timing and magnitude of expression in response to Zt infection. The pronounced early induction of *TaBON5*, *TaBON7*, and *TaBON9* was particularly notable, suggesting that these genes may play specialized roles in wheat’s early defense or interaction regulation against *Zt*, while other family members are likely involved in broader or sustained stress response pathways.

**Figure 8:**
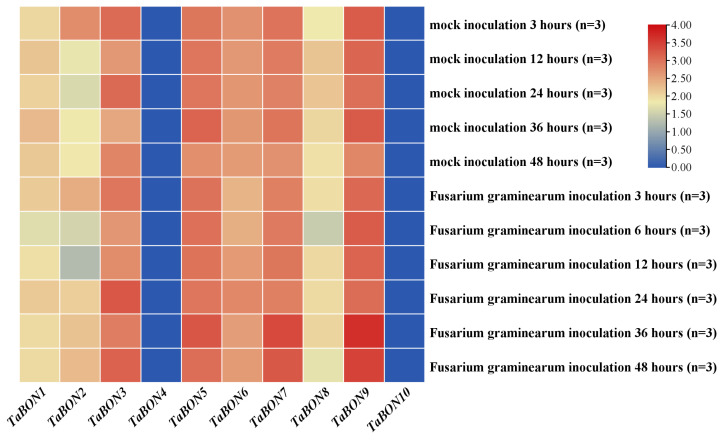
Heatmap of *TaBON* members expression under *Fusarium graminearum* stress from the ExpVIP database.

The expression patterns of the *TaBON* gene family in wheat following *Fusarium graminearum* infection were systematically analyzed. Compared to mock-treated controls, the majority of *TaBON* members exhibited no significant expression changes across the observed time points (Figure 8). For instance, *TaBON6* and *TaBON7* maintained stable transcript levels throughout the infection period, while *TaBON9* showed only marginal fluctuations at later stages (e.g., 36 hpi), with no statistically significant differences from the control group. Notably, the overall expression profiles of *TaBON* genes in pathogen-inoculated plants closely mirrored those in mock-treated conditions, suggesting that the *TaBON* family may not be directly involved in the early immune response to *F. graminearum* infection. These results indicate a limited functional diversification among *TaBON* members during this specific host–pathogen interaction, contrasting with their previously reported roles in other stress responses.

**Figure 9:**
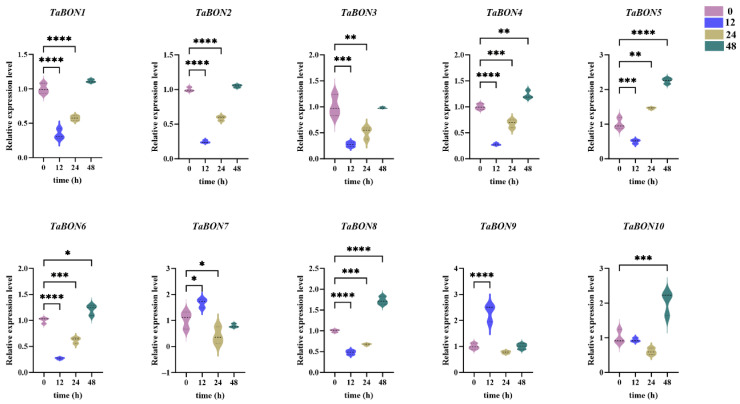
Gene expression patterns of ten *TaBON* members under powdery mildew stress (E09). *, **, ***, and **** indicate the significant correlations at the levels of *p* < 0.05, *p* < 0.01, *p* < 0.001, *p* < 0.0001, respectively.

Upon infection with the powdery mildew isolate E09, all *TaBON* genes displayed significant alterations in expression levels. At 12 h post-inoculation (hpi), the expression of all other *TaBON* genes was significantly down-regulated except for *TaBON7* and *TaBON9*. In contrast, only *TaBON5* exhibited clear time-dependent upregulation, indicating a specific response to fungal infection. Specifically, *TaBON5* expression was significantly induced at 24 hpi (*p* < 0.01), reached its peak at 48 hpi. *TaBON9* exhibited the most pronounced response, showing strong induction at 12 hpi (*p* < 0.0001), suggesting its potential role during the early stages of pathogen infection. A similar expression pattern was observed for *TaBON6*, *TaBON8* and *TaBON10*, which showed marked upregulation at 48 hpi (*p* < 0.001) (Figure 9). Overall, the *TaBON* gene family members were activated at different stages of pathogen infection, suggesting a complex and finely tuned temporal regulatory function in plant defense responses.

**Figure 10:**
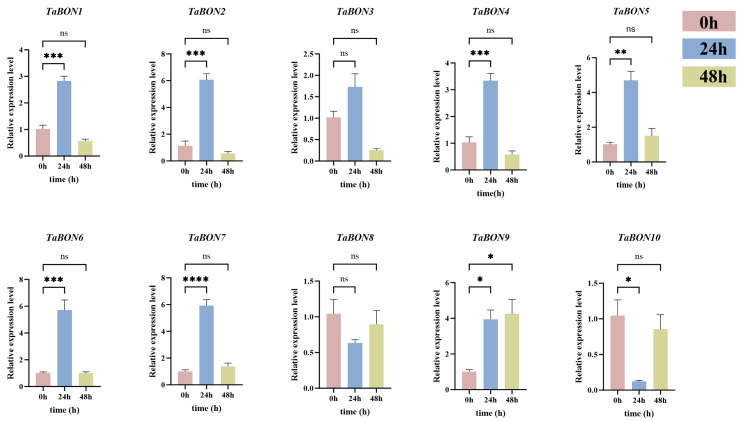
Differential expression of *TaBON* genes in response to stripe rust infection (CYR34). *, **, ***, and **** indicate the significant correlations at the levels of *p* < 0.05, *p* < 0.01, *p* < 0.001, and *p* < 0.0001 respectively, “ns” indicate no significant difference.

To further elucidate the expression regulation patterns of *TaBON* genes during stripe rust responses, the transcriptional dynamics of 10 *TaBON* genes in wheat were analyzed via RT-qPCR following inoculation with the fungus CYR34 at 0, 24, and 48 h post-inoculation. The results revealed that a total of seven *TaBON* genes were significantly upregulated at 24 hpi, with most peaking at 24 hpi and maintaining high expression levels or showing a decline by 48 hpi, demonstrating a typical pathogen-induced expression pattern. In contrast, a few genes, such as *TaBON3* and *TaBON8*, exhibited no significant changes in expression across all three time points, suggesting a weak response to pathogen infection. Notably, *TaBON10* was significantly downregulated at 24 hpi, indicating a distinct regulatory trend. These expression differences likely reflect functional diversification among *TaBON* gene family members within immune signaling pathways, with persistently highly expressed genes potentially playing important roles in the establishment or maintenance of defense responses (Figure 10).

Overall, *TaBON* gene family members may contribute to wheat immune responses through temporally distinct expression patterns and varying degrees of regulation, potentially fulfilling roles such as early defense, sustained resistance, or pathogen-specific adaptation during interactions with different pathogens. These findings provide important clues for further elucidating the mechanisms of wheat disease resistance and for identifying potential targets in breeding strategies.

### 3.7. Subcellular Localization of TaBON6 Protein

**Figure 11:**
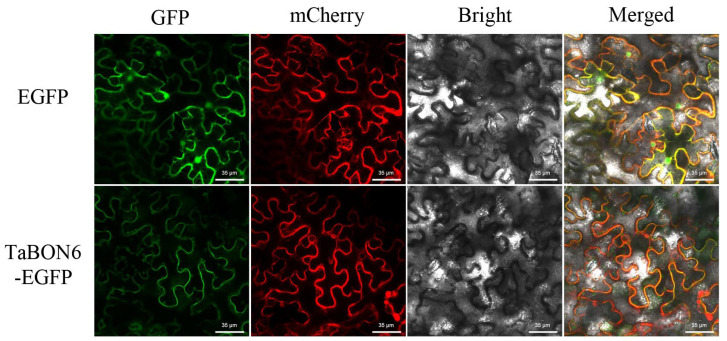
Subcellular localization of TaBON6 protein. Scale bars, 35 μm.

To gain deeper insights into the subcellular localization of TaBON6,we employed the transient expression of TaBON6-EGFP in *Nicotiana benthamiana* leaf cells. Although bioinformatic prediction suggested cytoplasmic and nuclear distribution, confocal microscopy revealed that the TaBON6-EGFP fusion protein was predominantly localized to the plasma membrane (Figure 11).

## 4. Discussion

### 4.1. Divergent Fates of Genes in Polyploid Evolution

The polyploidization events experienced by wheat provide a unique perspective for studying gene family evolution [31,32]. The absence of collinearity between the *TaBON* gene family and its *Arabidopsis* homologs may stem primarily from the substantial evolutionary divergence between dicotyledonous (*Arabidopsis*) and monocotyledonous (wheat, rice, etc.) plants, as well as from marked differences in genome structure and evolutionary history between the two species. As a hexaploid species, wheat possesses a large and complex genome formed through polyploidization, and has undergone extensive genomic rearrangements and lineage-specific gene expansion during evolution, which have disrupted the ancestral gene order. The uneven distribution of *TaBON* genes across the A, B, and D subgenomes, particularly the absence of homologs on chromosomes 7B and 7D, suggests the occurrence of gene loss after polyploidization [33,34]. This finding raises an important question: is this asymmetric distribution random or influenced by natural selection? We speculate that *TaBON* genes retained on specific chromosomes may have been under selective pressure due to their functional specificity. For instance, *TaBON* genes located on different subgenomes may have undergone differentiation in expression patterns or regulatory mechanisms, leading to functional complementarity or specialization [35]. This divergence in gene fate provides a typical case for understanding the evolution of gene families in polyploid crops.

### 4.2. Diversity in Cis-Regulatory Elements Suggests New Mechanisms of Functional Differentiation

Promoter analysis revealed the complexity in the regulatory mechanisms of *TaBON* genes. The combinatorial differences in cis-element composition in the promoter regions of different members, particularly the varying configurations of elements related to hormone response and stress adaptation, suggest that each member may participate in distinct biological processes through differential regulation [36]. Beyond revealing a complex landscape of cis-regulatory elements within the *TaBON* gene family, our analysis points to a deeper, testable hypothesis regarding functional evolution, proposing that the functional divergence of *TaBON* paralogs is largely driven by divergence in their transcriptional regulation. Specifically, we hypothesize that the unique combination of cis-elements in each promoter determines its responsiveness to specific internal or external cues, thereby partitioning family members into distinct biological processes such as defense versus development, while variation in stress-responsive element suites directly underlies observed pathogen-specific expression patterns, and differences in core or lineage-specific regulatory elements may contribute to the subgenome-biased expression of certain *TaBON* members. Promoter-reporter assays using mutagenesis of key elements can dissect their contribution to expression patterns, while comparative transcriptomics under different stresses can correlate element presence with induction profiles, thereby moving from correlation to causality in understanding *TaBON* regulation.

### 4.3. Expression Patterns Reveal Diversity in Pathogen Defense Strategies

Two copies of *TaBON1*, located on chromosomes 6AS and 6BL, correspond to *TaBON5* and *TaBON7* in the present study. With the continuous development of sequencing technology, an additional copy of *TaBON1* has been identified on wheat chromosome 6DS, which corresponds to *TaBON9* in this study. Three copies of *TaBON3*, distributed on chromosomes 1AL, 1BL, and 1DL respectively, correspond to *TaBON1*, *TaBON2*, and *TaBON3* in the present study. The distinct expression patterns of *TaBON* genes in response to different fungal pathogens suggest that wheat may employ targeted defense strategies by regulating the expression of specific *TaBON* members [16,37]. This expression specificity leads to a new hypothesis: members of the *TaBON* gene family might form a regulatory network through functional division of labor, enabling wheat to activate the most appropriate immune response based on the characteristics of different pathogen infections [38]. This finding not only deepens our understanding of the complexity of plant immune regulation but also provides a theoretical basis for designing precise disease resistance breeding strategies targeting specific pathogens [29]. Defining the precise regulatory mechanisms of TaBON members requires further functional validation.

### 4.4. From Gene Family Evolution to Crop Breeding Applications

The systematic analysis of the *TaBON* gene family in this study lays a functional genomics foundation for elucidating the molecular mechanisms underlying TaBON-mediated regulation of wheat disease resistance, and provides important genetic resources for wheat disease resistance breeding. The significant induction of several TaBON genes during early pathogen infection suggests that they may be key components in initial pathogen recognition [39]. However, owing to the lack of stable genetic transformation and direct functional validation in the present study, our current understanding is mainly derived from expression patterns and bioinformatic analyses. Thus, their precise positions in immune signaling pathways, interactions with other known resistance genes, and regulatory roles of post-translational modifications remain to be verified experimentally [40]. Further functional investigations, including VIGS and CRISPR/Cas9 mutagenesis, are currently underway in our laboratory to obtain direct functional evidence. These ongoing studies will help to establish the exact biological roles of TaBON genes in wheat–pathogen interactions and support more reliable applications in future wheat molecular breeding [41].

## 5. Conclusions

In this study, a comprehensive genome-wide analysis of the BON gene family in hexaploid wheat led to the identification of 10 TaBON genes. Phylogenetic and structural analyses revealed their evolutionary conservation and divergence, while expression profiling demonstrated their distinct responses to various fungal pathogens. These findings provide preliminary insights into the potential biological functions of TaBON genes during wheat–pathogen interactions, and suggest that these genes may serve as candidate resources for future research on wheat disease resistance. The functional divergence inferred among TaBON members implies a complex regulatory network associated with wheat defense responses, which may offer a useful reference for further exploring the molecular mechanisms underlying disease resistance in this globally important crop.

In this study, a comprehensive genome-wide analysis of the *BON* gene family in hexaploid wheat led to the identification of 10 *TaBON* genes. Phylogenetic and structural analyses revealed their evolutionary conservation and divergence, while expression profiling demonstrated their distinct responses to various fungal pathogens. These findings not only provide a fundamental resource for understanding the biological roles of *TaBON* genes in wheat immunity but also highlight their potential as candidate targets for breeding broad-spectrum disease-resistant varieties. The functional differentiation observed among *TaBON* members suggests a complex regulatory network underlying wheat–pathogen interactions, offering new avenues for deciphering the molecular basis of disease resistance in this globally critical crop.

## Figures and Tables

**Table 1:** Physicochemical parameters of *TaBON* members.

Name	Number of Amino Acid	Molecular Weight	Theoretical pI	Instability Index	Aliphatic Index	Grand Average of Hydropathicity	Subcellular Localization
*TaBON1*	590	64,936.66	5.68	33.89	85.27	−0.185	membrane
*TaBON2*	590	65,012.74	5.73	34.61	85.93	−0.186	membrane
*TaBON3*	590	64,798.46	5.76	35.02	85.1	−0.193	membrane
*TaBON4*	633	69,248.63	5.78	40.44	84.28	−0.173	membrane
*TaBON5*	574	63,103.65	5.23	34.63	90.02	−0.135	membrane
*TaBON6*	590	64,961.70	5.52	38.21	84.12	−0.189	membrane
*TaBON7*	577	63,222.64	5.23	36.66	87.89	−0.149	membrane
*TaBON8*	590	64,853.49	5.52	38.98	83.63	−0.211	membrane
*TaBON9*	574	62,960.44	5.18	34.89	89.34	−0.139	membrane
*TaBON10*	588	64,970.55	8.63	29.78	93.2	−0.122	membrane

## Data Availability

The original contributions presented in this study are included in the article. Further inquiries can be directed to the first author.

## Supplementary Materials

The following supporting information can be downloaded at: https://www.mdpi.com/article/10.3390/biology15090704/s1, Table S1: Protein sequence information of *TaBON* genes identified in this study; Table S2: Phylogenetic analysis of the *BON* gene family across five plant species; Table S3: Ratio of Ka/Ks and distribution of duplicate *TaBON* genes. Table S4: Characteristics of *cis*-acting regulatory elements in the promoter region of the identified *TaBON* genes.

## Abbreviations

The following abbreviations are used in this manuscript:

RT-qPCR	Reverse Transcription Quantitative Polymerase Chain Reaction
GRAVY	Grand Average of Hydropathy
pI	isoelectric points
ABA	abscisic acid
MBS	Matrix Binding Site
LTR	Long Terminal Repeat
MRE	Metal Response Element
MBSI	Matrix Binding Site I
GFP	Green Fluorescent Protein
CLSM	confocal laser scanning microscopy

## Appendix A

**Table A1:** Primer sequences used in this study.

Gene	Forward Primer Sequence	Reverse Primer Sequence
*TaBON1*	CCAGCGGGTTTGAGCTCA	TTGGTCTGCCAGAGGGGT
*TaBON2*	GCAGGTCACCGTTGTCCA	ACAGCTGGGGAGGAGGAG
*TaBON3*	TGCGGACTTCGGTAAGCG	TGGACAACGGTGACCTGC
*TaBON4*	GTCGGGGGTGCTGACTTC	GCCCACCTGCTTGGACTT
*TaBON5*	TGGGGAACTGCTGCTGTG	ATCAGCCACGGTGGAAGC
*TaBON6*	TGCGGACTTGGGTAAGCG	ACAGCTGGGGAGGAGGAG
*TaBON7*	CTCTGACGAGATGGGCGC	GGAGCGGAGGAAGCGATC
*TaBON8*	TGCGGACTTGGGTAAGCG	GCCCACCTGCTTGGACTT
*TaBON9*	GCTCTGACGAGATGGGCG	GGAGCGGAGGAAGCGATC
*TaBON10*	GGGCAGCTCGTGGAGTAC	TGCTGCCAAACACACCCA
*TaActin*	ACCTTCAGTTGCCCAGCAAT	CAGAGTCGAGCACAATACCAGTTG
